# Surge effects and survival to hospital discharge in critical care patients with COVID-19 during the early pandemic: a cohort study

**DOI:** 10.1186/s13054-021-03504-w

**Published:** 2021-02-17

**Authors:** Christopher R. Dale, Rachael W. Starcher, Shu Ching Chang, Ari Robicsek, Guilford Parsons, Jason D. Goldman, Andre Vovan, David Hotchkin, Tyler J. Gluckman

**Affiliations:** 1 Swedish Health Services, 600 Broadway, Suite 610, Seattle, WA 98122 USA; 2 School of Public Health, University of Washington, Seattle, WA USA; 3 Providence Portland Medical Center, Portland, OR USA; 4 Center for Cardiovascular Analytics, Research and Data Science (CARDS), Providence Heart Institute, Providence St. Joseph Health, Portland, OR USA; 5 Providence St. Joseph Health, Renton, WA USA; 6 Swedish Health Services, Seattle, WA USA; 7 Providence Health & Services, Portland, OR USA; 8 The Oregon Clinic, Portland, OR USA

**Keywords:** COVID-19, Critical care, Outcomes, Healthcare delivery, Surge effects, Health services

## Abstract

**Background:**

The early months of the COVID-19 pandemic were fraught with much uncertainty and some resource constraint. We assessed the change in survival to hospital discharge over time for intensive care unit patients with COVID-19 during the first 3 months of the pandemic and the presence of any surge effects on patient outcomes.

**Methods:**

Retrospective cohort study using electronic medical record data for all patients with laboratory-confirmed COVID-19 admitted to intensive care units from February 25, 2020, to May 15, 2020, at one of 26 hospitals within an integrated delivery system in the Western USA. Patient demographics, comorbidities, and severity of illness were measured along with medical therapies and hospital outcomes over time. Multivariable logistic regression models were constructed to assess temporal changes in survival to hospital discharge during the study period.

**Results:**

Of 620 patients with COVID-19 admitted to the ICU [mean age 63.5 years (SD 15.7) and 69% male], 403 (65%) survived to hospital discharge and 217 (35%) died in the hospital. Survival to hospital discharge increased over time, from 60.0% in the first 2 weeks of the study period to 67.6% in the last 2 weeks. In a multivariable logistic regression analysis, the risk-adjusted odds of survival to hospital discharge increased over time (biweekly change, adjusted odds ratio [aOR] 1.22, 95% CI 1.04–1.40, *P* = 0.02). Additionally, an a priori-defined explanatory model showed that after adjusting for both hospital occupancy and percent hospital capacity by COVID-19-positive individuals and persons under investigation (PUI), the temporal trend in risk-adjusted patient survival to hospital discharge remained the same (biweekly change, aOR 1.18, 95% CI 1.00–1.38, *P* = 0.04). The presence of greater rates of COVID-19 positive/PUI as a percentage of hospital capacity was, however, significantly and inversely associated with survival to hospital discharge (aOR 0.95, 95% CI 0.92–0.98, *P* < 0.01).

**Conclusions:**

During the early COVID-19 pandemic, risk-adjusted survival to hospital discharge increased over time for critical care patients. An association was also seen between a greater COVID-19-positive/PUI percentage of hospital capacity and a lower survival rate to hospital discharge.

## Background

The coronavirus 2019 (COVID-19) global pandemic caused by SARS-CoV-2 has posed significant challenges to intensive care units (ICUs) across the world. Initial reports from China noted 83% survival in hospitalized patients, 61% in ICU patients, and only 3% in patients receiving mechanical ventilation [1, 2]. Similar findings were reported in the Western USA, where investigators in the Seattle area initially reported approximately 50% survival in ICU patients [3, 4].

During the early months of the COVID-19 pandemic in the USA and Europe, many organizations treated “surges” of patients during a time of incomplete understanding of critical illness related to SARS-CoV-2 and a lack of clearly effective COVID-19-specific therapies. Reported data sets in initial publications were often incomplete, with half or more patients missing hospital outcomes [5–7]. Pre-print servers like MedRxIV became common and frequently shared sources of information on prognosis, treatments, and outcomes. Social media reports related to drug therapy evolved rapidly over the first several months of the pandemic [8–10]. Early on, there was much interest in therapies for hypoxemic respiratory failure, with discussion around the utility of delaying or minimizing intubation, using or not using noninvasive positive pressure ventilation and when to use high-flow oxygen [11, 12]. In addition to the ambiguous and evolving clinical situation, resource constraints, rationing in some areas of the world and discussions of crisis care standards, may have each contributed to adverse patient outcomes [13–15]. Nonetheless, some studies were able to demonstrate improved survival to hospital discharge during this early period [16, 17].

With critical care resources (e.g., ventilators, nurses, respiratory therapists, and ICU beds) at the center of crisis planning, we were interested in better understanding temporal changes in outcomes for critical care patients with COVID-19 during the first several months of the pandemic. We hypothesized that survival to hospital discharge increased during this time and sought to evaluate this within a large integrated delivery system in the Western USA. Additionally, we hypothesized that volume-related “surge effects,” including stress on hospital personnel and resources, may have adversely impacted survival to hospital discharge.

## Methods

### Study setting and data collection

We conducted an observational cohort study of all COVID-19 patients, admitted to an ICU for at least 6 h, across twenty-six hospitals in a large healthcare system in the Western USA from February 25, 2020, to May 15, 2020. Data were collected through May 28, 2020, with exclusion of patients that remained admitted at the end of the study period and/or were readmitted. For simplicity of display, patient characteristics and outcomes were summarized in 2-week cohorts by the date of admission for bivariate analysis, with assessment of differences between and across the cohorts. As part of our multivariable analysis, we modelled admissions in 1-week intervals for greater precision and have reported results on a per-week basis, starting with the first week of the study.

Persons under investigation (PUIs) were defined as those in whom a COVID-19 PCR test had been sent, but not yet resulted. We were interested in the effect of increased COVID-19/PUI census on the hospital as a whole and modeled the surge effects of increased hospital volumes in two ways. First, hospital occupancy was defined as inpatient census on the day of admission divided by total number of licensed hospital beds. Second, COVID-19-positive/PUI percent hospital capacity was similarly calculated by dividing the sum of PUI and COVID-19-positive patients on the day of patient admission by the total number of licensed hospital beds. The day of admission was chosen to summarize volume effects for clarity and reproducibility.

Patient data including socio-demographics, comorbidities, severity of illness, COVID-19 treatments, laboratory results, and outcomes data were obtained from the electronic medical record (Epic Systems, Madison, WI). Median household income was imputed from patient’s home ZIP code based on 2006–2010 US Census data. The Sequential Organ Failure Assessment (SOFA) score was calculated based on the most severe values obtained in the first 24 h of hospitalization. Exposures to drug and oxygen therapies were measured as binary variables based on any exposure to the therapy. Prone positioning was based on review of medical record documentation.

### Statistical analysis

We used descriptive statistics to assess the distribution of all variables of interest. Continuous variables are presented as means or median as appropriate, and categorical variables are presented as frequencies. Comparisons between groups were performed by ANOVA or Kruskal–Wallis rank-sum tests for continuous variables, and Chi-squared or Fisher exact tests for categorical variables. If there was significant difference between groups, Jonckheere–Terpstra tests and Chi-squared tests for trend were used to evaluate for temporal trends (ordered differences) across sequential biweekly time periods for continuous and categorical variables, respectively. To determine the temporal pattern and independent factors for survival to hospital discharge, we used multivariable logistic regression analyses, with admission date modeled on a continuous basis. The odds ratios with corresponding 95% confidence intervals were presented. Variables including time of admission, age, gender, body mass index (BMI), race, income, smoking status, marital status, comorbidities, and SOFA scores were evaluated and selected for adjusted regression based on the literature, a priori associations, and bivariate associations. The linearity of the continuous variables in relation to the logit of the binary outcome (discharged alive, yes vs no), and final model diagnosis were assessed for linearity assumptions and the overall agreement between observed and fitted values.

Robustness of observed temporal trends from the final multivariable model was evaluated with a hierarchical random effects model to account for hospital-level cluster effects. In our a priori explanatory model on surge effects, which was estimated by percentage of hospital beds filled with COVID-19-positive/PUI patients, survival to hospital discharge was evaluated. We also further evaluated whether the temporal trend in risk-adjusted patient survival to hospital discharge was explained by the trend in the hospital occupancy and the COVID/PUI percentage capacity, both individually in the multivariable model and then together with the same set of covariates as in our main model for risk-adjusted survival to hospital discharge. This project was approved by the Swedish Health Services Institutional Review Board. Statistical analyses were performed in R, version 3.6.3 (R Core Team 2020).

## Results

### Description of the study cohort

#### Patients

During the study period, 650 patients with COVID-19 were admitted: 13 (2.0%) patients were excluded for subsequent readmissions and 17 (2.7%) were excluded for continued hospitalization at the end of the data collection period. Among the 620 patients included in our analysis, the mean age was 63.5 (SD 15.7) years, 430 (69.4%) were male, 267 (43.1%) were White, and 181 (29.2%) were Hispanic or Latinx (Table 1). Temporal trends in patient characteristics are reported in Table 1. The median BMI was 28.2 kg/m^2^ (IQR 24.3–33.1) and 31 (5.0%) described themselves as current users of tobacco. A total of 303 (48.9%) patients described themselves as married, and the mean household median annual adjusted gross income in the patient’s home zip code was $68,943 (SD $18,019).

**Table 1:** Demographic and clinical characteristics of patients

	Overall (*n* = 620)	First period (*n* = 35)	Second period (*n* = 168)	Third period (*n* = 164)	Fourth period (*n* = 125)	Fifth period (*n* = 94)	Sixth period (*n* = 34)	*P *value
Date range	Mar 01–May 15	Mar 01–Mar 14	Mar 15–Mar 28	Mar 29–April 11	April 12–April 25	April 26–May 09	May 10–May 15	
Hospital occupancy (%)^a^, median, [interquartile range (IQR)]	54.5 (45.0–63.4)	70.7 (63.8–85.8)	55.35 (44.1–61.9)	48.1 (41.7–55.0)	53.7 (43.0–61.1)	59.4 (52.8–65.6)	61.7 (51.1–72.4)	< 0.001
COVID-19-positive/PUI census (%)^b^, median (IQR)	9.8 (6.6–14.8)	4.7 (1.2–7.7)	10.5 (8.0–14.9)	12.0 (8.2–15.7)	9.2 (5.6–16.7)	8.1 (3.9–14.3)	7.2 (4.1–9.6)	< 0.001
COVID-19-positive census (%)^c^, median (IQR)	4.6 (1.7–7.7)	0.4 (0.0–2.1)	2.1 (0.8–4.5)	5.6 (3.3–8.2)	6.3 (3.4–10.7)	5.8 (2.7–10.7)	4.9 (1.6–6.8)	< 0.001
Age (years), mean [standard deviation (SD)]	63.5 (15.7)	63.7 (15.7)	63.1 (14.6)	63.4 (15.5)	65.8 (14.7)	61.9 (17.2)	61.7 (19.6)	0.514
Male sex, *n* (%)	430 (69.4)	21 (60.0)	119 (70.8)	124 (75.6)	83 (66.4)	58 (61.7)	25 (73.5)	0.155
BMI, median (IQR)	28.2 (24.3–33.1)	28.3 (23.5–32.5)	30.0 (25.8–34.1)	27.2 (23.6–31.2)	28.1 (24.4–33.9)	27.2 (22.9–32.5)	28.7 (24.3–35.3)	0.002
Race/ethnicity, *n* (%)								0.012
White or Caucasian	267 (43.1)	22 (62.9)	88 (52.4)	54 (32.9)	53 (42.4)	36 (38.3)	14 (41.2)	
Asian	53 (8.5)	5 (14.3)	13 (7.7)	15 (9.1)	9 (7.2)	10 (10.6)	1 (2.9)	
Black or African-American	31 (5.0)	0 (0.0)	11 (6.5)	11 (6.7)	6 (4.8)	2 (2.1)	1 (2.9)	
Hispanic or Latino	181 (29.2)	3 (8.6)	37 (22.0)	52 (31.7)	41 (32.8)	34 (36.2)	14 (41.2)	
Other/unknown	88 (14.2)	5 (14.3)	19 (11.3)	32 (19.5)	16 (12.8)	12 (12.8)	4 (11.8)	
Median income, mean (SD)	68,943.4 (18,019.3)	60,106.7 (18,987.3)	66,875.7 (17,983.1)	67,368.2 (16,857.2)	71,623.9 (17,064.6)	76,244.6 (18,939.9)	65,814.1 (16,360.8)	< 0.001
Smoking status, *n* (%)								0.170
Never smoker	368 (59.4)	21 (60.0)	96 (57.1)	102 (62.2)	71 (56.8)	56 (59.6)	22 (64.7)	
Current smoker	31 (5.0)	1 (2.9)	6 (3.6)	7 (4.3)	10 (8.0)	6 (6.4)	1 (2.9)	
Former smoker	174 (28.1)	12 (34.3)	60 (35.7)	41 (25.0)	32 (25.6)	20 (21.3)	9 (26.5)	
Unknown	47 (7.6)	1 (2.9)	6 (3.6)	14 (8.5)	12 (9.6)	12 (12.8)	2 (5.9)	
Marital status, *n* (%)								0.005
Not married	279 (45.0)	13 (37.1)	67 (39.9)	62 (37.8)	65 (52.0)	52 (55.3)	20 (58.8)	
Married	303 (48.9)	22 (62.9)	94 (56.0)	88 (53.7)	53 (42.4)	33 (35.1)	13 (38.2)	
Unknown	38 (6.1)	0 (0.0)	7 (4.2)	14 (8.5)	7 (5.6)	9 (9.6)	1 (2.9)	
Hypertension, *n* (%)	195 (31.5)	12 (34.3)	50 (29.8)	45 (27.4)	41 (32.8)	36 (38.3)	11 (32.4)	0.588
Diabetes, *n* (%)	174 (28.1)	9 (25.7)	41 (24.4)	51 (31.1)	31 (24.8)	33 (35.1)	9 (26.5)	0.420
Chronic kidney disease, *n* (%)	71 (11.5)	4 (11.4)	13 (7.7)	14 (8.5)	23 (18.4)	7 (7.4)	10 (29.4)	0.001
Coronary artery disease, *n* (%)	53 (8.5)	5 (14.3)	13 (7.7)	12 (7.3)	11 (8.8)	7 (7.4)	5 (14.7)	0.588
Congestive heart failure, *n* (%)	48 (7.7)	5 (14.3)	13 (7.7)	6 (3.7)	11 (8.8)	11 (11.7)	2 (5.9)	0.138
Chronic obstructive pulmonary disease (COPD), *n* (%)	43 (6.9)	3 (8.6)	11 (6.5)	6 (3.7)	13 (10.4)	8 (8.5)	2 (5.9)	0.341
Asthma, *n* (%)	32 (5.2)	4 (11.4)	11 (6.5)	4 (2.4)	9 (7.2)	4 (4.3)	0 (0.0)	0.108
Cirrhosis, *n* (%)	9 (1.5)	1 (2.9)	1 (0.6)	3 (1.8)	2 (1.6)	2 (2.1)	0 (0.0)	0.802
End-stage renal disease (ESRD), *n* (%)	17 (2.7)	0 (0.0)	2 (1.2)	4 (2.4)	6 (4.8)	3 (3.2)	2 (5.9)	0.319
Total comorbidities, *n* (%)								0.026
0	277 (44.7)	10 (28.6)	74 (44.0)	74 (45.1)	63 (50.4)	40 (42.6)	16 (47.1)	
1	163 (26.3)	10 (28.6)	52 (31.0)	52 (31.7)	19 (15.2)	24 (25.5)	6 (17.6)	
2 +	180 (29.0)	15 (42.9)	42 (25.0)	38 (23.2)	43 (34.4)	30 (31.9)	12 (35.3)	
Initial Sequential Organ Failure Assessment (SOFA) Score, mean (SD)	3.97 (2.99)	3.34 (2.63)	3.61 (3.04)	4.33 (3.02)	4.16 (2.79)	3.72 (2.95)	4.62 (3.48)	0.104
World Health Organization (WHO) score at admissio*n* (%)								0.001
2	14 (2.3)	0 (0.0)	3 (1.8)	2 (1.2)	4 (3.2)	5 (5.3)	0 (0.0)	
3	133 (21.5)	10 (28.6)	38 (22.6)	30 (18.3)	23 (18.4)	26 (27.7)	6 (17.6)	
4	293 (47.3)	18 (51.4)	85 (50.6)	80 (48.8)	56 (44.8)	35 (37.2)	19 (55.9)	
5	62 (10.0)	3 (8.6)	6 (3.6)	10 (6.1)	24 (19.2)	17 (18.1)	2 (5.9)	
6	33 (5.3)	3 (8.6)	10 (6.0)	10 (6.1)	5 (4.0)	3 (3.2)	2 (5.9)	
7	82 (13.2)	1 (2.9)	26 (15.5)	31 (18.9)	13 (10.4)	6 (6.4)	5 (14.7)	
8	3 (0.5)	0 (0.0)	0 (0.0)	1 (0.6)	0 (0.0)	2 (2.1)	0 (0.0)	

^a^Inpatient census on the day of admission/total number of licensed hospital beds × 100

^b^Sum of COVID-19-positive and PUI censuses on the day of patient admission/total number of licensed hospital beds × 100

^c^Sum of COVID-19-positive censuses on the day of patient admission/total number of licensed hospital beds × 100

By past medical history or problem list entry, 195 (31.5%) patients had hypertension, 174 (28.1%) had diabetes, and 71 (11.5%) had chronic kidney disease of any stage; 277 (44.7%) had no medical comorbidities. The mean initial 24-h SOFA score was 4.0 (SD 3.0). Additional patient details are summarized by 2-week cohort in Table 1.

#### Treatments

Three hundred and twenty-one (51.8%) received hydroxychloroquine, but the percentage was not evenly distributed across the 2-week cohorts (*P* < 0.01), with 127 (75.6%) of patients receiving hydroxychloroquine in the second 2-week cohort in March 2020 and only 4 (11.8%) in the final cohort in May 2020. One hundred and nine (17.6%) patients received remdesivir with significant difference across the 2-week cohorts (*P* = 0.03), with 4 (11.4%) receiving remdesivir in the first 2-week cohort and 6 (17.6%) in the final 2-week cohort.

Five hundred and seven (81.8%) patients were on room air at some point in their hospitalization, and 529 (85.3%) patients received nasal cannula at some point, and neither therapy differed significantly by 2-week cohort (*P* = 0.3 for both). Three hundred and seventy-one (59.8%) patients underwent invasive mechanical ventilation, with 23 (65.7%) patients during the first 2-week cohort and significantly decreasing to 14 (41.2%) by the final 2-week cohort (*P* < 0.01 for the trend). Additionally, 296 (47.7%) patients received high-flow nasal cannula. There was a significant increase in the use of high-flow oxygen from the first 2-week cohort (10 patients, 28.6%) to the final cohort (18 patients, 52.9%; *P* < 0.01). Additional treatment details are found in Table 2.

**Table 2:** Treatments and (ICU) LOS by 2-week cohorts

	Overall (n = 620)	First period (n = 35)	Second period (n = 168)	Third period (n = 164)	Fourth period (n = 125)	Fifth period (n = 94)	Sixth period (n = 34)	*P *value
Date range	Mar 01–May 15	Mar 01–Mar 14	Mar 15–Mar 28	Mar 29–April 11	April 12–April 25	April 26–May 09	May 10–May 15	
Hydroxychloroquine, *n* (%)	321 (51.8)	15 (42.9)	127 (75.6)	99 (60.4)	59 (47.2)	17 (18.1)	4 (11.8)	< 0.001
Remdesivir, *n* (%)	109 (17.6)	4 (11.4)	31 (18.5)	33 (20.1)	11 (8.8)	24 (25.5)	6 (17.6)	0.029
Tocilizumab, *n* (%)	88 (14.2)	1 (2.9)	22 (13.1)	27 (16.5)	23 (18.4)	11 (11.7)	4 (11.8)	0.220
Dexamethasone, *n* (%)	28 (4.5)	0 (0.0)	10 (6.0)	6 (3.7)	7 (5.6)	4 (4.3)	1 (2.9)	0.655
Room air, *n* (%)	507 (81.8)	31 (88.6)	145 (86.3)	133 (81.1)	95 (76.0)	75 (79.8)	28 (82.4)	0.260
Nasal cannula, *n* (%)	529 (85.3)	32 (91.4)	150 (89.3)	141 (86.0)	103 (82.4)	75 (79.8)	28 (82.4)	0.252
Mask, *n* (%)	411 (66.3)	25 (71.4)	119 (70.8)	105 (64.0)	79 (63.2)	60 (63.8)	23 (67.6)	0.676
Continuous positive airway pressure (CPAP), *n* (%)	78 (12.6)	6 (17.1)	22 (13.1)	23 (14.0)	13 (10.4)	12 (12.8)	2 (5.9)	0.709
Bi-level positive airway pressure (BIPAP), *n* (%)	87 (14.0)	4 (11.4)	24 (14.3)	16 (9.8)	20 (16.0)	17 (18.1)	6 (17.6)	0.448
High-flow nasal cannula, *n* (%)	296 (47.7)	10 (28.6)	56 (33.3)	86 (52.4)	76 (60.8)	50 (53.2)	18 (52.9)	< 0.001
Invasive mechanical ventilation, *n* (%)	371 (59.8)	23 (65.7)	131 (78.0)	98 (59.8)	65 (52.0)	40 (42.6)	14 (41.2)	< 0.001
Prone ventilation, *n* (%)	356 (57.4)	16 (45.7)	97 (57.7)	97 (59.1)	87 (69.6)	47 (50.0)	12 (35.3)	0.002
Duration of invasive mechanical ventilation (days), median (IQR)	9.1 (4.7–14.4)	8.6 (3.5–17.0)	9.9 (7.0–15.4)	7.4 (4.1–12.1)	8.9 (3.9–15.9)	8.7 (4.0–15.7)	3.9 (2.5–9.6)	0.018
ICU length of stay (LOS) (days), median (IQR)	6.2 (2.7–12.5)	4.8 (3.4–13.7)	9.1 (3.5–13.6)	6.0 (2.5–11.4)	6.2 (2.3–14.1)	5.0 (2.6–9.1)	4.0 (1.5–7.4)	0.004
Hospital LOS (days), median (IQR)	12.7 (7.5–21.8)	10.7 (7.1–28.4)	14.1 (8.1–23.0)	13.6 (8.3–22.1)	13.4 (7.2–21.6)	10.0 (5.9–17.02)	8.7 (5.5–16.1)	0.009

#### Outcomes

**Fig. 1:**
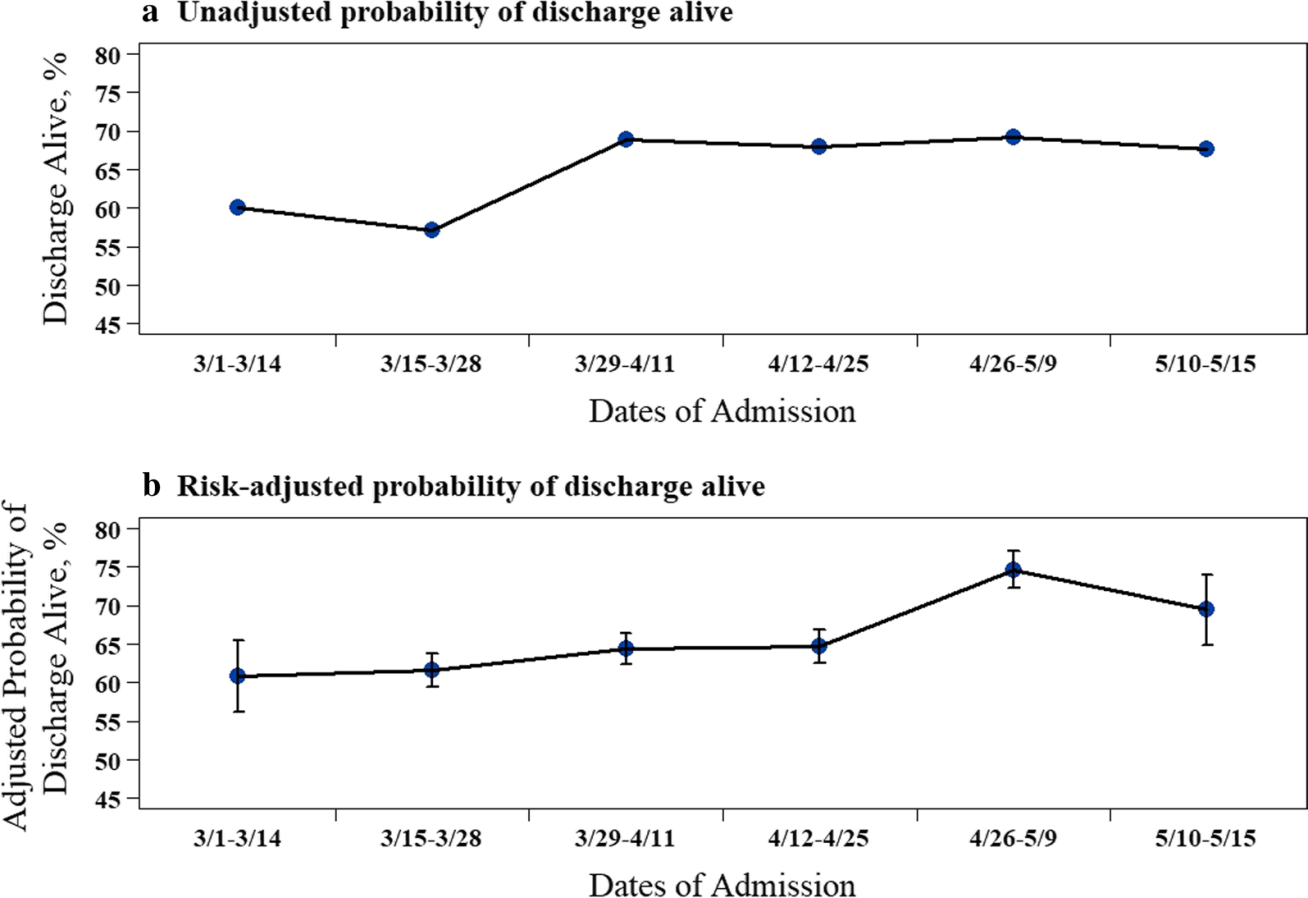
Survival to hospital discharge over time during the initial months of the COVID-19 pandemic for critical care patients with COVID-19. **a** Unadjusted proportion of patients surviving to hospital discharge. **b** Risk-adjusted probability of surviving to hospital discharge. Error bars represent the standard errors (SE) of the point estimates

Overall, 403 (65.0%) patients were discharged alive, increasing from 60.0% during the first 2 weeks of the study period to 67.6% in the last 2 weeks (Fig. 1a). Of the 217 patients who died during their hospitalization, 176 (81.1% of deaths) occurred in the ICU. Of those patients treated with invasive mechanical ventilation, median (IQR) time on the ventilator was 9.1 (4.7–14.4) days, with a noted temporal decrease across the 2-week cohorts (*P* = 0.02). Median time in the ICU and hospital was 6.2 (2.7–12.5) days and 12.7 (7.5–21.8) days, respectively, and both varied significantly by 2-week cohort (*P* < 0.01 and *P* = 0.01, respectively). Additional outcomes are shown in Table 2.

### Trend and predictors of survival to hospital discharge

#### Univariate analysis

As shown in Table 3, the odds of being discharged alive increased over time, not adjusting for other covariates (biweekly change, OR 1.14, 95% CI 1.00 to 1.28, *P* = 0.04). Year of age (OR 0.94, 95% CI 0.93–0.95, *P* < 0.01) and smoking status (OR 0.51, 95% CI 0.35–0.74, *P* < 0.01) were both associated with decreased odds of survival to hospital discharge. Asian (OR 1.98, 95%CI 1.03–3.83, *P* = 0.04) and Hispanic/Latinx (OR 1.63, 95% CI 1.09–2.43, *P* = 0.02) race/ethnicity were associated with greater odds of survival to hospital discharge than White/Caucasian. Household median income based on home ZIP code was associated with increased survival to hospital discharge ($1000 change, OR 1.02, 95% CI 1.01–1.03, *P* < 0.01). Hospital occupancy was inversely associated with survival to hospital discharge (OR 0.98, 95% CI 0.97–0.99, *P* < 0.01). COVID-positive/PUI percent hospital capacity was also inversely associated with survival to hospital discharge (OR 0.94, 95% CI 0.92–0.97, *P* < 0.01).

**Table 3:** Multivariable logistic regression analyses for independent predictors associated with survival to hospital discharge

	Univariate analysis (*n* = 620)				Multivariable analysis				Multivariable analysis*			
	OR	Lower 95% CI	Upper 95% CI	*P* value	OR	Lower 95% CI	Upper 95% CI	*P* value	OR	Lower 95% CI	Upper 95% CI	*P* value
Admission date (biweekly)	1.07	1.00	1.14	0.042	1.11	1.02	1.20	0.017	1.09	1.00	1.19	0.049
Age (years)	0.94	0.93	0.95	< 0.001	0.92	0.90	0.93	< 0.001	0.91	0.89	0.93	< 0.001
Male sex (reference: female)	0.92	0.64	1.32	0.648	0.88	0.55	1.40	0.596	0.90	0.56	1.47	0.687
BMI	0.98	0.96	1.01	0.157	0.93	0.90	0.96	< 0.001	0.93	0.90	0.96	< 0.001
Race and ethnicity (reference: White or Caucasian)												
Asian	1.98	1.03	3.83	0.041	1.46	0.66	3.21	0.349	1.24	0.53	2.91	0.619
Black or African American	0.86	0.41	1.83	0.702	0.67	0.28	1.63	0.378	0.58	0.23	1.46	0.243
Hispanic or Latino	1.63	1.09	2.43	0.017	0.65	0.38	1.10	0.108	0.69	0.39	1.23	0.207
Other/unknown	2.01	1.18	3.43	0.010	1.69	0.85	3.37	0.136	1.74	0.84	3.59	0.135
Median gross income ($1000)	1.02	1.01	1.03	< 0.001	1.02	1.01	1.04	< 0.001	1.02	1.00	1.04	0.112
Smoking status (reference: never smoker)												
Current smoker	1.23	0.53	2.82	0.633	1.13	0.4	3.19	0.819	1.18	0.40	3.50	0.764
Former smoker	0.51	0.35	0.74	< 0.001	0.74	0.47	1.18	0.208	0.73	0.45	1.19	0.206
Unknown	0.58	0.31	1.07	0.081	0.7	0.32	1.51	0.365	0.72	0.32	1.64	0.436
Marital status (reference: not married)												
Married	1.23	0.87	1.72	0.241	1.49	0.97	2.3	0.067	1.50	0.95	2.38	0.081
Unknown	1.72	0.8	3.67	0.165	0.84	0.33	2.18	0.727	0.88	0.32	2.40	0.804
Hypertension (reference: no)	0.83	0.58	1.18	0.297	1.14	0.72	1.81	0.583	1.21	0.74	2.00	0.449
Diabetes (reference: no)	0.81	0.56	1.16	0.253	0.82	0.51	1.32	0.422	0.84	0.50	1.38	0.485
Chronic kidney disease (reference: no)	0.58	0.35	0.96	0.033	1.49	0.79	2.81	0.223	1.56	0.80	3.03	0.188
Coronary artery disease (reference: no)	0.88	0.49	1.57	0.663	1.28	0.62	2.63	0.499	1.18	0.56	2.52	0.659
Congestive heart failure (reference: no)	0.46	0.26	0.84	0.011	0.70	0.34	1.44	0.338	0.78	0.37	1.65	0.523
COPD (reference: no)	0.6	0.32	1.11	0.104	0.90	0.41	2.00	0.804	0.90	0.39	2.09	0.800
Asthma (reference: no)	1.98	0.84	4.67	0.1162	0.90	0.32	2.56	0.848	0.764	0.263	2.222	0.621
Initial SOFA score	0.83	0.78	0.88	< 0.001	0.83	0.77	0.89	< 0.001	0.820	0.760	0.885	< 0.001

*Mixed effects logistic regression model including admission time, baseline patient characteristics as fixed effects, and a hospital indicator variable as a random effect

### Multivariable analysis

In the final multivariable logistic regression model, the odds of being discharged alive increased over time throughout the study period, after adjusting for age, gender, BMI, race, income, smoking status, marital status, hypertension, diabetes, chronic kidney disease, coronary artery disease, congestive heart failure, COPD, asthma, and SOFA scores (biweekly change, aOR 1.22, 95% CI 1.04–1.420, *P* = 0.02, Table 3). On average, the risk-adjusted patient survival increased from 60.8% (first 2 weeks) to 69.5% (last 2 weeks) over the study period (Fig. 1b). This finding held true after accounting for hospital-level random effects (biweekly change, aOR 1.18, 95% CI 1.00–1.38, *P* = 0.049). Other significant predictors of survival to hospital discharge include greater household median income ($1000 change, aOR 1.02, 95% CI 1.01–1.04, *P* < 0.01), age (yearly change, aOR 0.92, 95% CI 0.90–0.94, *P* < 0.01), and BMI (one-unit change, aOR 0.93, 95% CI 0.90–0.96, *P* < 0.01).

To estimate the mean causal mediation effects on trends in patient survival to discharge, we further applied the causal mediation effects method with quasi-Bayesian Monte Carlo approximation using statistical R package mediation at both patient level and hospital level, accounting for hospital clustering effects to estimate the mean causal mediation effects of invasive mechanical ventilation on trends in patient survival to discharge [18–20]. The same set of covariates was adjusted in both mediator and outcome models.

In our a priori-defined explanatory models, greater hospital occupancy and higher COVID-positive/PUI percent hospital capacity were each inversely associated with survival to hospital discharge (aOR 0.98, 95% CI 0.97–1.00, *P* = 0.04 and aOR 0.94, 95% CI 0.92–0.97, *P* < 0.01, respectively). After adjusting for both hospital occupancy and COVID-positive/PUI percent hospital capacity and the same set of covariates as in the primary model, the temporal trend in risk-adjusted patient survival to hospital discharge remained the same (biweekly change, aOR 1.18, 95% CI 1.00–1.38, *P* = 0.04). In this model, hospital occupancy was not independently associated with survival to hospital discharge (*P* = 0.3); however, COVID-positive/PUI percent hospital capacity remained significantly inversely associated with survival to hospital discharge (1% increase, aOR 0.95, 95% CI 0.92–0.98, *P* < 0.01).

## Discussion

In our cohort of COVID-19 patients admitted to the ICU, survival to hospital discharge increased over time. To our knowledge, this is one of the most complete reporting of outcomes for this population. Of note, patients included were similar in age and sex to those reported previously, were more likely to be Hispanic, and had a lower burden of many comorbidities, including hypertension and coronary artery disease [5, 7, 21–25]. Findings from this study support the previously reported inverse association of age and BMI with survival to hospital discharge [7, 23, 26]. In addition, the association between median household income and COVID-19 outcomes has also been reported and may reflect access to care or biases in care delivery [27].

The exact mechanism by which each week was associated with increased survival to hospital discharge is not clear from our study. Changes in evidence-based therapies over time seem unlikely to have played a significant role. Despite early promising data and initial enthusiasm for hydroxychloroquine, later trials and meta-analyses have failed to demonstrate a benefit [28–32]. Significant participation in early remdesivir trials likely underlies the 17.6% of patients who received remdesivir, which is higher than reported elsewhere [6, 7]. However, changes in remdesivir biweekly are unlikely to have caused the improved survival to hospital discharge, as later trials and a meta-analysis have failed to demonstrate improved survival to hospital discharge, especially among ICU patients [32, 34, 36]. An increase in the use of steroids over time could have contributed to increased survival, but our study predated much of the clinical trial data for steroids, with low overall use.

Similar to a lack of obvious change in medical therapy driving increased survival to hospital discharge over time, changes in oxygen and respiratory therapy were also unlikely to have played a prominent role. The use of invasive mechanical ventilation decreased over time in our cohort as the use of high-flow oxygen increased. Initial reports emphasized an early-intubation strategy for COVID-19, a strategy that ultimately proved controversial and has not been associated with improved outcomes [1, 5, 11, 12]. Over the study period, evidence emerged about the benefits of high-flow oxygen and non-intubated prone positioning, but despite effects on oxygenation, these therapies also have not been associated with improved survival [37–39]. Similarly, noninvasive positive pressure ventilation did not vary throughout the study period, and its use has not been definitively associated with improved survival [40].

Our secondary hypothesis focused on “surge effects” and the idea that high-volume stressors on care delivery could have driven the observed increase in survival over time. In our explanatory model, the percentage of hospital beds occupied by COVID-19-positive/PUI patients was independently and inversely associated with survival to hospital discharge. Diagnostic and therapeutic uncertainly early into the COVID-19 pandemic was associated with very high ICU mortality, especially in mechanically ventilated patients [1, 3, 41]. In fact, it was uncertain early into the pandemic if our hospitals would have enough resources to care for all affected patients. In March 2020, Italian hospitals developed strategies for rationing limited resources in the face of models predicting a surge that would overstress their hospital system [13, 42, 43]. Our organization also developed scare resource triage plans based off of those created by the Northwest Healthcare Response Network in Washington State [15]. Ultimately, we did not have to enact scare resource triage, but the surge in COVID-19-positive patient volumes may have had an unmeasured effects on patient care that was more subtle. To that end, our preliminary internal data show numeric increases in catheter-associated urinary tract infections and central line bloodstream infection rates. The National Healthcare Surveillance Network data for healthcare associated infections will be able to shed more light on this question over time. Interestingly, overall hospital volumes were not independently associated with changes in survival to hospital discharge, independent of COVID-19/PUI volumes, which might speak to COVID-specific effects or more focused effects within the ICU, rather than hospital-wide effects.

In addition to any surge effect based on patient volume, the timing of the surge in our cohort toward the beginning of the pandemic may be relevant. In addition, newness of COVID-19 as a disease, unfamiliarity of care teams with its treatment, and the social milieu of the pandemic may have contributed to a volume-related “surge effect.” Unfortunately, our data are unable to address that difference.

At the start of the COVID-19 pandemic, the clinical course of the illness was uncertain, with early reports noting poor survivability, especially in elderly intubated patients [1]. Anecdotally, in some of our critical care teams, this led to discussions with patients and surrogates on earlier transitions to comfort-focused care than we would routinely recommend for other patients with acute respiratory distress syndrome (ARDS), critical illness or respiratory failure. As the biology and clinical course of COVID-19-associated respiratory compromise became more clear, providers may have reverted to anchoring on survivability estimates based on other patients with ARDS, with increased survival to hospital discharge. Further work is needed to move beyond these hypotheses and to separate early pandemic effects from surge effects and their respective independent contributions to hospital discharge.

Finally, in our exploratory analysis, the use of invasive mechanical ventilation significantly decreased the odds of survival to hospital discharge (OR 0.26, 95% CI 0.17–0.37, *P* < 0.01) and decreased significantly over time (biweekly change, OR 0.68, 95% CI 0.60–0.77, *P* < 0.01). We further applied the causal mediation analysis to estimate the mean effects of invasive mechanical ventilation on trends in patient survival to discharge. Mediation analysis showed that decreasing use of invasive mechanical ventilation was significantly associated with 1.4% and 1.3% increases in survival to hospital discharge in patient- and hospital-level analyses, respectively. This indicates that the increasing trend in risk-adjusted patient survival to discharge may be explained by decreasing use of invasive mechanical ventilation or an unmeasured covariate.

### Limitations

Our study has several important limitations. First, our cohort was limited to patients in the Western USA and may not be representative of other regions or countries. Thus, the generalizability of our learnings may be limited. Second, we focused our analysis on the initial months of the pandemic. As such, its extrapolation to other points in time may not be applicable. Third, our study looked at patients admitted to the ICU. We focused on the ICU as it has been a critical resource constraint in the pandemic and of great interest to the critical care community. However, it is possible that changes in the severity of illness among patients admitted to the ICU varied over time. While the fact that the SOFA score did not vary over time speaks against this, there could have been other unmeasured confounders. We did not discretely capture data on geographic expansion of ICU care into locations beyond traditional ICUs, which could be an additional explanatory variable. Fourth, we were limited by variables we can extract from the electronic medical record and were not able to explore daily patient counts or drug exposures with more subtlety. As such, it is possible that more refined variable collection could have provided additional information, including changes in delays from out-of-hospital symptom onset to presentation for hospital admission. Fifth, we chose to model surge effects in a linear manner. It is possible, however, that threshold effects or a nonlinear relationship exists between overall hospital volume or COVID-19/PUI volume. Finally, while we tested for independent associations between hospital occupancy percentage and COVID-positive/PUI percentage of hospital capacity in our explanatory models, our ability to fully explain the change in survival to hospital discharge over time is limited by the lack of more detailed data on behaviors of patients, families, providers, and care teams over time. In addition, imprecision in modeling the surge and simply summarizing volume on the day of admission may have limited the analysis. Furthermore, additional qualitative investigation is needed to address the mechanistic reasons for the observed changes, especially differentiating early pandemic effects from surge effects.

## Conclusions

Early into the COVID-19 pandemic, patients admitted to ICUs within a large integrated delivery system in the Western USA were noted to have improved survival to hospital discharge over time. An association between greater COVID-19/PUI volume and lesser survival to hospital discharge was also observed.

## Supplementary Information

**Additional file 1**. Supplementary multivariable models and patient flow diagram.

## Data Availability

The dataset used during the current study is available from the corresponding author on reasonable request.

## Abbreviations

ARDS: Acute respiratory distress syndrome
BIPAP: Bi-level positive airway pressure
BMI: Body mass index
COPD: Chronic obstructive pulmonary disease
CPAP: Continuous positive airway pressure
ESRD: End-stage renal disease
ICU: Intensive care unit
IQR: Interquartile range
PUI: Person under investigation
SD: Standard deviation
SOFA: Sequential Organ Failure Assessment
WHO: World Health Organization
