# Supplementary material for: Surge effects and survival to hospital discharge in critical care patients with COVID-19 during the early pandemic: a cohort study
Source: Crit Care. 2021 Feb 17;25:70. doi: 10.1186/s13054-021-03504-w (PMC7887411; doi:10.1186/s13054-021-03504-w)
Supplement: Supplementary file 1 — Additional file 1. Supplementary multivariable models and patient flow diagram. [file 13054_2021_3504_MOESM1_ESM.docx]

600 Broadway, Suite 610

Seattle, WA. 98122

January 4, 2021

*Critical Care*

Elie Azoulay, MD PhD, Associate Editor

Jean-Louis Vincent, MD PhD, Editor-in-Chief

Dear Prof. Elie Azoulay and Editorial Team:

We appreciate the opportunity to revise our manuscript, *Surge Effects and Survival to Hospital Discharge in Critical Care Patients with COVID-19 During the Early Pandemic: A Cohort Study*, and submit it to you for further consideration.

We appreciate points raised by the reviewer and the editorial staff and hope our revisions successfully address your concerns.

Our point-by-point response follows:

*Thank you for this submission. As pointed out by the reviewers, information is still lacking in the manuscript. For instance, too few patients have been admitted in such a large number of centres, there is no flow chart to show that the data are little biased, the analysis seems to have selected the variables to be included. Please consider that this is a major revision.* 

Thank you for your comments. We are limited by the number of patients treated in our hospitals. We do think that geographic diversity of our cohort, centered on the Seattle, Portland and Los Angeles makes the conclusions interesting.

We thought that a focused look at ICU patients would be relevant to a critical care audience. During the study period, the study hospitals provided care for 2211 patients with COVID-19, 650 of whom required ICU admission. Supplementary Figure 1 presents a flow diagram of the ICU inclusion/exclusion criteria.

We studied ICU utilization over time and included the SOFA score, as well as medical comorbidities and sociodemographic features in our final model to adjust for the possibility that severity of illness changed over time. Because ICU admission criteria vary between institution, we have included that as a limitation (Pages 16-17, Lines 406-8).

*Reviewer #1: In a large series of COVID-19 patients admitted in the ICU, Dale and colleagues retrospectively report that (i) prognostic improved between the beginning and the end of the wave and (ii) it was associated with parameters of hospitals "stress.”*

*While interesting, the manuscript and the analysis deserves a few comments.
One could be surprised that in 26 hospitals, during such a pandemic between 2 months, only 620 ICU patients were recorded. This regards around 24 critically-ill patients per hospital, which is a small number.*

*Could you give the number of ICUs as well as the total number of ICU beds? A flow-chart could be interesting to detect selection bias, if any. It is not obvious in the table 1, based on hospital occupancy, that hospitals were overbooked whatever the period. More, even if it was significantly different between the periods of time, it was much variable and we cannot observe a real decrease from the first period to the last period of time.*

Thank you for the comments. As most of these hospitals are smaller or medium-sized community hospitals, they only have one ICU. Seven have two ICUs. In terms of selection bias, the patient-selection criteria, as outlined in the manuscript (Pages 8 and 9) and in Supplementary Figure 1 (below), were relatively straight-forward and pulled directly from our electronic data warehouse. Possible selection bias could have occurred if there were systematic coding errors. However, we think that the risk of such bias is low because the datamart from which our study drew is the COVID-19 reporting system of record for the health system for this pandemic. As such, it is highly scrutinized and matches with actual hand counts of patients across our health care system.

In terms of times of stress on ICU care resulting in informal rationing of critical care resources, that certainly could be a possibility. We did attempt to control for severity of illness by adjusting for the SOFA score, sociodemographic features and medical comorbidities in our model. Though we did not have to formally triage patients beyond standard ICU admission practice, that “surge effects” could have resulted in adverse patient selection is a good point that have included in the discussion and limitations (Pages 16 and 17).

*Could you confirm that a COVID-19/PUI census (%) of 12% means that 12% of the ICU beds were occupied by COVID-19 patients? Thanks to clarify this point in the method.*

*It is especially unclear if number of COVID-19 patients (or suspicion) was divided by the total number of licensed beds or by the number of ICU beds. Moreover, I did not understand why the ICU occupancy was only focused on COVID-19 patients. What about the other patients? They also count in the occupancy.*

Thank you for bringing these points up, as they are important. The COVID-19 and PUI hospital census (%) does not reflect the percentage of ICU bed that were occupied by COVID, rather it is a hospital-level variable. We did not model ICU occupancy, either with or without COVID-19/PUI patients. When creating this variable to model strain on the system, we considered looking at ICU COVID census, and ultimately decided that because there is some variation in the decision to admit to the ICU, which may or may not be influenced by a patient’s COVID status, as well as sharing of resources throughout the hospital, that overall hospital COVID status was a better representation of strain within the hospital, which was the organization phenomenon we were attempting to capture. This may very well be subtly different than ICU strain, as we indicated in our discussion and we have added additional language to the introduction, methods and discussion to clarify this because it is an important point, and could well be a focus for the next step of this line of inquiry.

*Could you confirm that all the ICUs were already existing before the pandemic and that no de novo ICUs were created. If so, it should be taken into account as one may argue that prognosis would be different between both.* 

This is a great point about a possible mechanism for surge effects, the expansion of critical care beyond the walls of traditional ICUs. Given the relatively mild surge numbers seen in the US West Coast versus other areas of the world, the expansion of critical care into novel settings was very limited in our study cohort institutions. No de novo ICUs were created. We cannot rule-out that individual single patients included in the study were not cared for outside of traditional ICUs for periods of time, but our study inclusion criteria were to focus on patients in critical care status admitted to ICUs for > 6 hours. We have added language in the discussion to emphasize non-traditional ICUs as an explanatory variable in sure effects.

*About statistical analysis: it was already reported such a prognosis improvement along the pandemic in a large series of more than 4000 patients (see COVID-ICU, Intensive Care Med 2020). In this paper, the delay between symptoms onset and ICU admission was associated with prognosis, as a worst prognosis was observed when patients were admitted during the first week following symptoms onset. Do you have any ability to get such information into the statistical analysis?*

Yes, this is a great point and other groups are showing similar increases in survival over time.[1,2] While we did not capture the data of symptom onset in the dataset, we do have the data of admission to the hospital. If we add time from hospital admission to ICU admission as a continuous or categorical variable into the primary models, the significance and direction of the effects of the other variables remain the same (supplementary Table 1). This speaks against intrahospital changes in time to ICU admission as being a factor in the observed results, but we cannot comment on pre-hospital delays and we have added that caveat to the discussion and additional context to the introduction.

*I also wonder why you did not adjust on the need for invasive mechanical ventilation as it is a marker of severity but may also aggravate lung function and prognosis by itself. I think that you should. Thanks to clarify in the results that when you say that 59.8% of patients required mechanical ventilation you mean invasive or invasive plus non-invasive.*

Thank you for this suggestion. We didn’t include mechanical ventilation in our primary model because our primary objective was to assess the temporal trend in survival to hospital discharge. However, we agree that mechanical ventilation is an important marker of severity and as you say potentially in the causal pathway to duration of ICU stay. Our exploratory analysis did show a significant decreasing trend in mechanical ventilation usage, and hence further evaluated it in the framework of causal mediation analysis to further evaluate mean causal mediation effects of mechanical ventilation on trends in patient survival to discharge.[3–5] As you anticipated, mediation analysis showed that decreasing use of mechanical ventilation was significantly associated with a 1.4% and 1.3% increase in survival to hospital discharge at the patient and hospital-levels, respectively. We have added this to both the methods and discussion.

Finally, yes, 59.8% of patients required invasive mechanical ventilation and we have clarified this. Thank you for bringing it up.

*Finally, you should probably soften the conclusion as increased COVID-19/PUI volume did not explain but was associated with increased hospital discharge.*

Yes, thank you for pointing this out. We tried not to overstate the conclusion, but were using the word “explain” in the sense of “observed an association,” but based upon your suggestion, we changed the phrasing to decrease any confusion. Our observational study cannot address causation.

Thank you so much for considering our manuscript and providing valuable feedback. We hope that we have satisfactorily address your points and we look forward to hearing from you.

Sincerely,

Chris Dale, MD MPH

Chief Quality Officer

Pulmonary and Critical Care Medicine

Swedish Health Services

Clinical Assistant Professor

Health Services

University of Washington

[Christopher.dale@swedish.org](mailto:Christopher.dale@swedish.org)

425-747-5822

References:

1. Dennis JM, McGovern AP, Vollmer SJ, Mateen BA. Improving Survival of Critical Care Patients With Coronavirus Disease 2019 in England: A National Cohort Study, March to June 2020. Crit Care Med. 2020;Publish Ahead of Print.

2. Horwitz LI, Jones SA, Cerfolio RJ, Francois F, Greco J, Rudy B, et al. Trends in COVID-19 Risk-Adjusted Mortality Rates. J Hosp Med. 2020;

3. Lederer DJ, Bell SC, Branson RD, Chalmers JD, Marshall R, Maslove DM, et al. Control of Confounding and Reporting of Results in Causal Inference Studies. Guidance for Authors from Editors of Respiratory, Sleep, and Critical Care Journals. Ann Am Thorac Soc. 2018;16:22–8.

4. Bradley SM, Liu W, McNally B, Vellano K, Henry TD, Mooney MR, et al. Temporal Trends in the Use of Therapeutic Hypothermia for Out-of-Hospital Cardiac Arrest. Jama Netw Open. 2018;1:e184511.

5. Tingley D, Yamamoto T, Hirose K, Keele L, Imai K. mediation : R Package for Causal Mediation Analysis. J Stat Softw. 2014;59.

Supplementary Table 1. Multivariable logistic regression analyses for independent predictors associated with survival to hospital discharge.

|  | **Multivariable Analysis 1** | | | | **Multivariable Analysis 2** | | | |
| --- | --- | --- | --- | --- | --- | --- | --- | --- |
|  | **aOR** | **Lower 95% CI** | **Upper 95% CI** | **p-value** | **aOR** | **Lower 95% CI** | **Upper 95% CI** | **p-value** |
| **Admission date (bi-weekly)** | 1.22 | 1.03 | 1.44 | 0.020 | 1.22 | 1.04 | 1.44 | 0.018 |
| **Days from Admission to ICU** | 0.94 | 0.89 | 1 | 0.044 |  |  |  |  |
| **Days from Admission to ICU (Reference ≤7 days)** |  |  |  |  |  |  |  |  |
| 8-14 days |  |  |  |  | 0.93 | 0.35 | 2.50 | 0.888 |
| > 14 days |  |  |  |  | 0.13 | 0.02 | 0.98 | 0.048 |
| **Age (years)** | 0.92 | 0.90 | 0.93 | <0.001 | 0.91 | 0.90 | 0.93 | <0.001 |
| **Male Sex (Reference: Female)** | 0.89 | 0.56 | 1.42 | 0.619 | 0.88 | 0.55 | 1.41 | 0.602 |
| **BMI** | 0.93 | 0.90 | 0.96 | <0.001 | 0.93 | 0.90 | 0.96 | <0.001 |
| **Race and Ethnicity (Reference: White or Caucasian)** |  |  |  |  |  |  |  |  |
| Asian | 1.42 | 0.64 | 3.15 | 0.383 | 1.49 | 0.67 | 3.33 | 0.331 |
| Black or African American | 0.67 | 0.28 | 1.62 | 0.374 | 0.67 | 0.28 | 1.62 | 0.377 |
| Hispanic or Latino | 0.68 | 0.40 | 1.16 | 0.154 | 0.66 | 0.39 | 1.12 | 0.126 |
| Other/Unknown | 1.80 | 0.90 | 3.60 | 0.099 | 1.74 | 0.87 | 3.47 | 0.118 |
| **Median Gross Income ($1,000)** | 1.02 | 1.01 | 1.04 | <0.001 | 1.02 | 1.01 | 1.04 | <0.001 |
| **Smoking Status (Reference: Never Smoker)** |  |  |  |  |  |  |  |  |
| Current Smoker | 1.12 | 0.40 | 3.16 | 0.831 | 1.12 | 0.40 | 3.18 | 0.825 |
| Former Smoker | 0.75 | 0.47 | 1.19 | 0.216 | 0.74 | 0.46 | 1.17 | 0.198 |
| Unknown | 0.70 | 0.32 | 1.53 | 0.376 | 0.68 | 0.31 | 1.48 | 0.331 |
| **Marital Status (Reference: Not married)** |  |  |  |  |  |  |  |  |
| Married | 1.45 | 0.94 | 2.23 | 0.092 | 1.47 | 0.95 | 2.26 | 0.080 |
| Unknown | 0.80 | 0.31 | 2.07 | 0.640 | 0.81 | 0.31 | 2.09 | 0.656 |
| **Hypertension (Reference: No)** | 1.19 | 0.75 | 1.90 | 0.457 | 1.20 | 0.75 | 1.91 | 0.450 |
| **Diabetes (Reference: No)** | 0.81 | 0.5 | 1.31 | 0.390 | 0.83 | 0.52 | 1.34 | 0.458 |
| **Chronic Kidney Disease (Reference: No)** | 1.48 | 0.78 | 2.81 | 0.228 | 1.47 | 0.77 | 2.80 | 0.241 |
| **Coronary Artery Disease (Reference: No)** | 1.35 | 0.65 | 2.79 | 0.418 | 1.31 | 0.63 | 2.70 | 0.470 |
| **Congestive Heart Failure (Reference: No)** | 0.70 | 0.34 | 1.43 | 0.326 | 0.71 | 0.34 | 1.45 | 0.345 |
| **COPD (Reference: No)** | 0.95 | 0.43 | 2.11 | 0.905 | 0.97 | 0.44 | 2.14 | 0.933 |
| **Asthma (Reference: No)** | 0.92 | 0.32 | 2.61 | 0.875 | 0.88 | 0.31 | 2.52 | 0.818 |
| **Initial SOFA Score** | 0.82 | 0.76 | 0.88 | <0.001 | 0.83 | 0.77 | 0.89 | <0.001 |
|  | | | | | | | | |

Supplementary Figure 1. Study inclusion/exclusion criteria.
